# Data supporting mitochondrial morphological changes by SPG13-associated HSPD1 mutants

**DOI:** 10.1016/j.dib.2015.12.038

**Published:** 2016-01-04

**Authors:** Yuki Miyamoto, Funakoshi-Tago Megumi, Nanami Hasegawa, Takahiro Eguchi, Akito Tanoue, Hiroomi Tamura, Junji Yamauchi

**Affiliations:** a Department of Pharmacology, National Research Institute for Child Health and Development, Setagaya, Tokyo 157-8535, Japan; b Faculty of Pharmacy, Keio University, Minato, Tokyo 105-8512, Japan; c Graduate School of Medical and Dental Sciences, Tokyo Medical and Dental University, Bunkyo, Tokyo 113-8510, Japan

**Keywords:** SPG13, HSPD1, Mitochondrion, Morphological change

## Abstract

The data is related to the research article entitled “Hypomyelinating leukodystrophy-associated missense mutation in HSPD1 blunts mitochondrial dynamics” [1]. In addition to hypomyelinating leukodystrophy (HLD) 4 (OMIM no. 612233), it is known that spastic paraplegia (SPG) 13 (OMIM no. 605280) is caused by HSPD1’s amino acid mutation. Two amino acid mutations Val-98-to-Ile (V98I) and Gln-461-to-Glu (Q461E) are associated with SPG13 [2]. In order to investigate the effects of HSPD1’s V98I or Q461E mutant on mitochondrial morphological changes, we transfected each of the respective mutant-encoding genes into Cos-7 cells. Either of V98I or Q461E mutant exhibited increased number of mitochondria and short length mitochondrial morphologies. Using MitoTracker dye-incorporating assay, decreased mitochondrial membrane potential was also observed in both cases. The data described here supports that SPG13-associated HSPD1 mutant participates in causing aberrant mitochondrial morphological changes with decreased activities.

## Specifications Table

**Table:** 

Subject area	Biology
More specific subject area	Cell biology, Biochemistry
Type of data	Figure
How data was acquired	Cytochemistry
Data format	Raw and analyzed data
Experimental factors	Mitochondrial morphologies in SPG13-associated HSPD1 mutant-introduced Cos-7 cells
Experimental features	Fluorescent and confocal microscopic analysis
Data source location	National Research Institute for Child Health and Development, Tokyo, Japan
Data accessibility	Data is available with this study

## **Value of the data**

• Disease-associated HSPD1 mutant-expressing mitochondria exhibited short length morphologies.
• Disease-associated HSPD1 mutant-expressing mitochondria increased their number in cells.
• Disease-associated HSPD1 mutant-expressing mitochondria exhibit their decreased membrane potential in MitoTracker dye-incorporating assay.

## 1 Data, experimental design, materials and methods

### 1.1 Experimental design

To explore how spastic paraplegia (SPG13)-associated mutant of HSPD1 has an effect on mitochondrial morphology, we set out to study transfection experiments using Cos-7 cells. Since this cell line has large cytoplasmic regions, it is suitable to observe morphological changes of mitochondria [1].

### 1.2 Plasmid constructs

Gene recombinations were carried out in accordance with a protocol approved by the Japanese National Research Institute for Child Health and Development Gene Recombination Committee. The cDNA encoding HSPD1 was amplified from total human brain cDNAs (NipponGene, Tokyo, Japan). The cDNA encoding SPG13-associated Val-98-to-Ile (V98I) or Gln-461-to-Glu (Q461E) mutant of HSPD1 [2] was produced by the method of overlapping PCR. They were ligated into the *Bam*HI multiple cloning site (MCS) of the mammalian expression vector pTagGFP (Evrogen, Moscow, Russia). As the result, their plasmids encoded fusion proteins possessing six amino acids in MCS plus TagGFP in the C-terminus of HSPD1 protein. DNA sequences were confirmed by Fasmac (Kanagawa, Japan). The pTagRFP-Mito (mitochondrially-localized RFP) plasmid was purchased from Evrogen. TagGFP and TagRFP are monomeric green and red fluorescent proteins, respectively.

### 1.3 Cell culture and transfection

Monkey kidney Cos-7 cells (Human Science BioResource Bank, Tokyo, Japan) were cultured on cell culture dishes (Greiner Bio-One, Oberösterreich, Germany) in DMEM containing 10% heat-inactivated FBS and PenStrep (Thermo Fisher Scientific, Waltham, MA, USA) in 5% CO_2_ at 37 °C. Cells were transfected with plasmids using a Lipofectamine LTX Plus transfection kit (Thermo Fisher Scientific) or a CalPhos transfection kit (Takara Bio, Kyoto, Japan) according to the respective manufacturers’ instructions. The medium was replaced 4 h after transfection for the lipofection method or 18 h after transfection for the calcium phosphate method and transfected cells were used for experiments 48 h after transfection [3], [4].

### 1.4 Cytochemical studies

**Fig. 1:**
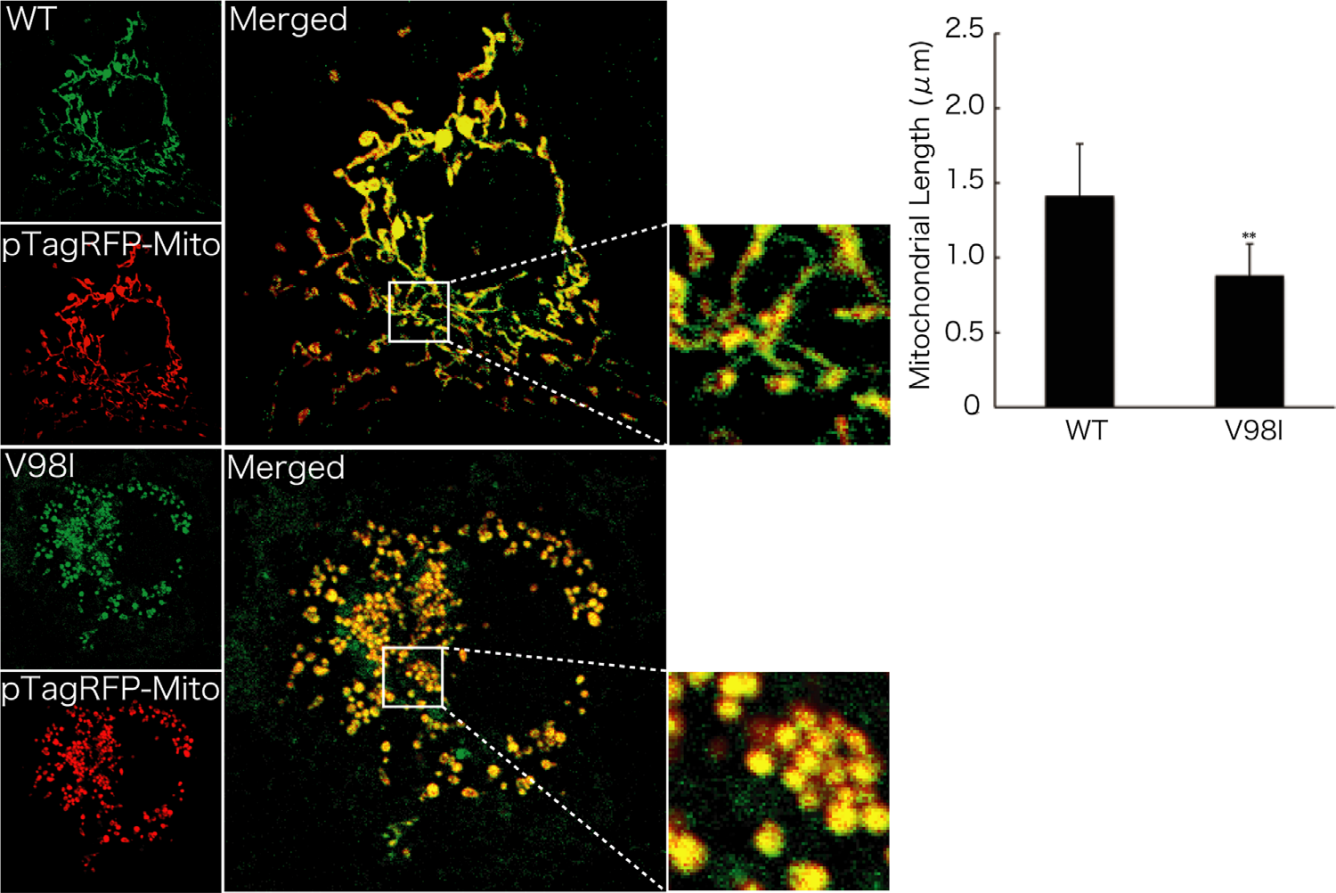
Mitochondria harboring HSPD1 (V98I) exhibit short length phenotypes. Cells were transfected with the pTagRFP-mitochondrion-localized Mito plasmid (red fluorescence) together with the pTagGFP-wild type (WT) HSPD1 or pTagGFP-HSPD1 (V98I) (green fluorescence) plasmid. The merged photograph (yellow fluorescence) and the high magnification image indicated by white square are shown. The length of mitochondria is also shown in the graph (**, *p*<0.05; *n*=70 mitochondria from 3 cells of the respective independent experiments).

**Fig. 2:**
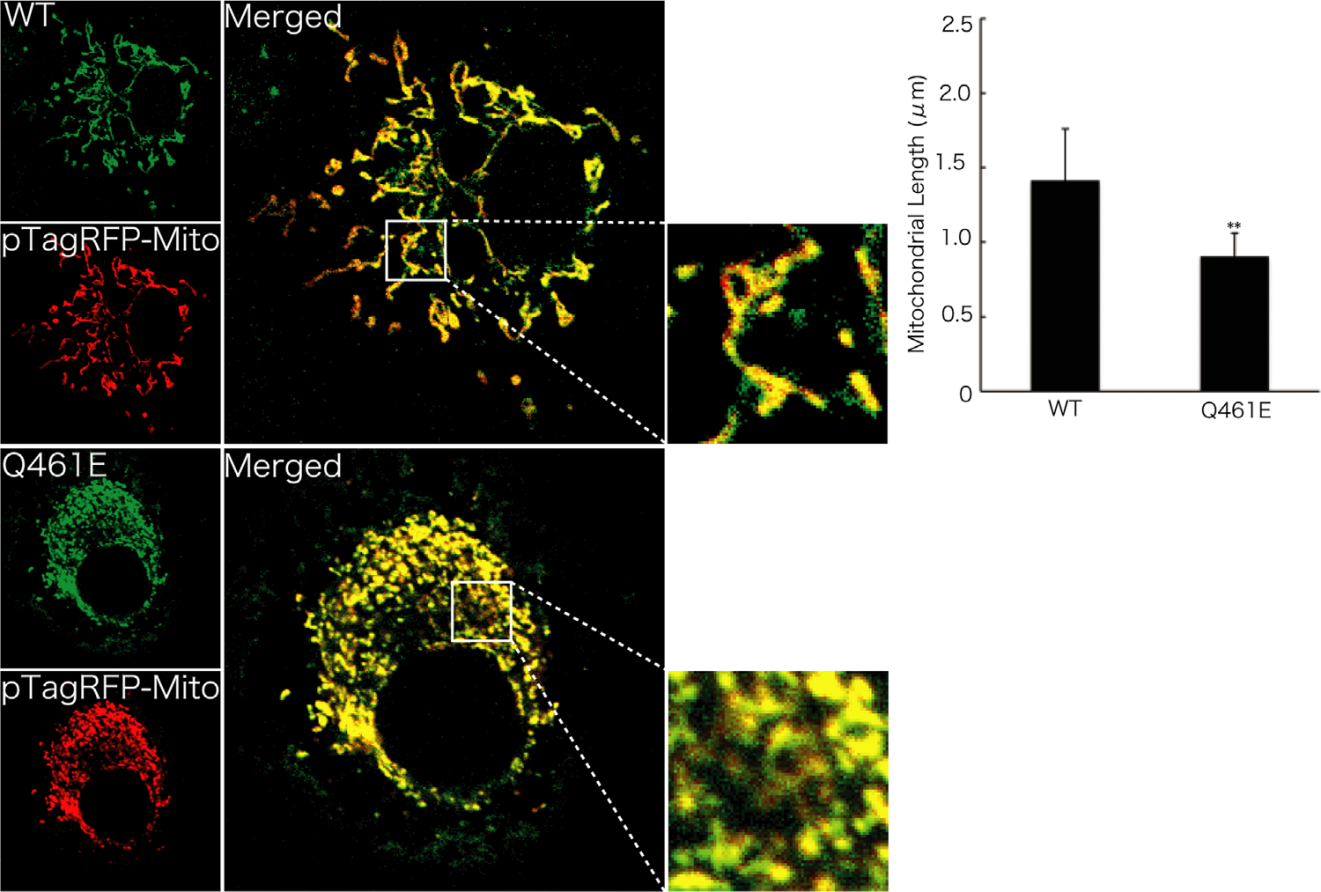
Mitochondria harboring HSPD1 (Q461E) exhibit short length phenotypes. Cells were transfected with the pTagRFP-Mito plasmid (red fluorescence) together with the pTagGFP-wild type (WT) HSPD1 or pTagGFP-HSPD1 (Q461E) plasmid (green fluorescence). The merged photograph (yellow fluorescence) and the high magnification image indicated by white square are shown. The length of mitochondria is also shown in the graph (**, *p*<0.05; *n*=70 mitochondria from 3 cells of the respective independent experiments).

**Fig. 3:**
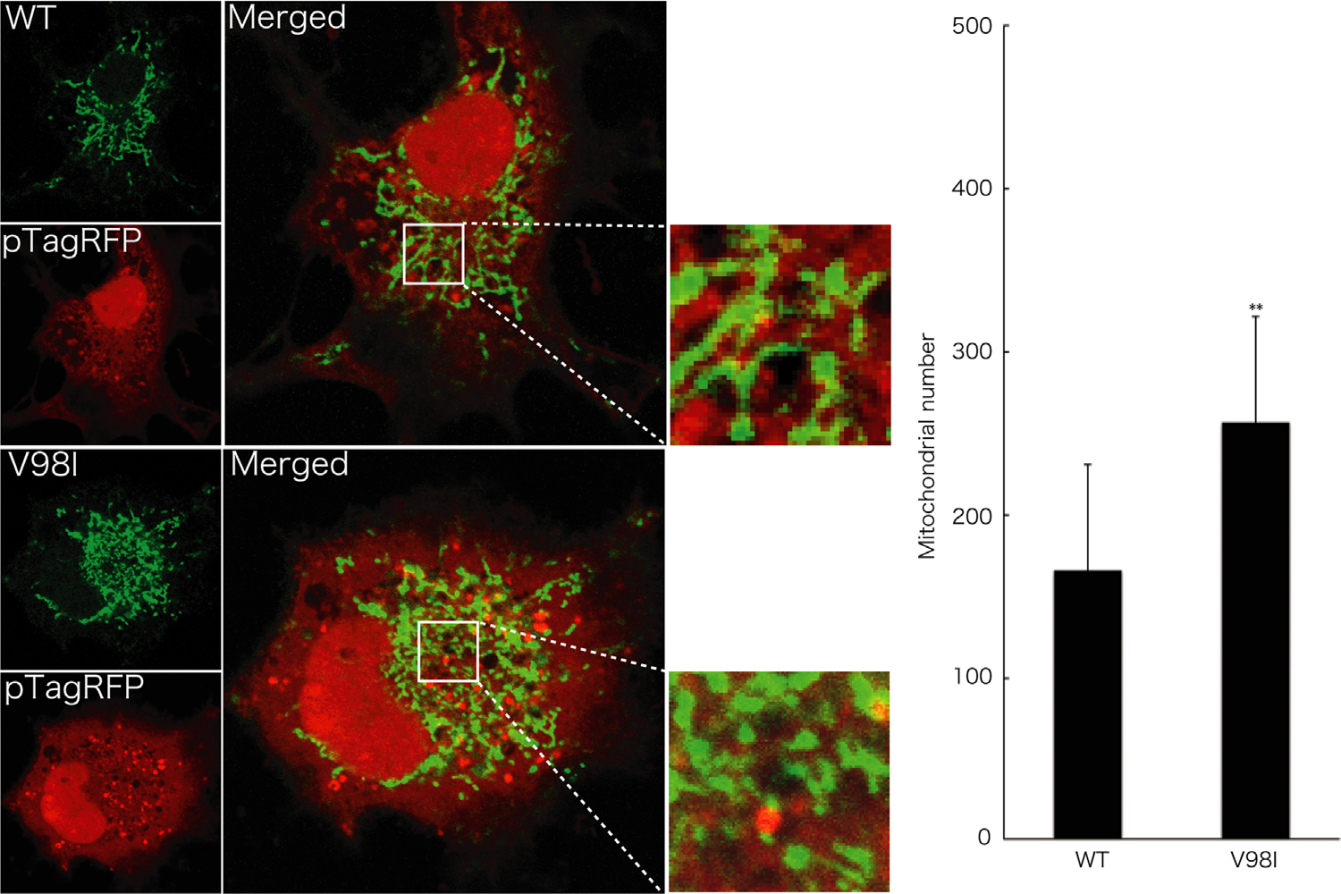
The number of mitochondria harboring HSPD1 (V98I) is greater than that of mitochondria harboring wild type HSPD1. Cells were transfected with the empty vector pTagRFP (red fluorescence), to mark whole cytoplasmic regions, together with the plasmid encoding wild type (WT) HSPD1 or HSPD1 (V98I) (green fluorescence). The number of mitochondria is also shown in the graph (**, *p*<0.05; *n*=12 cells from 3 independent experiments).

**Fig. 4:**
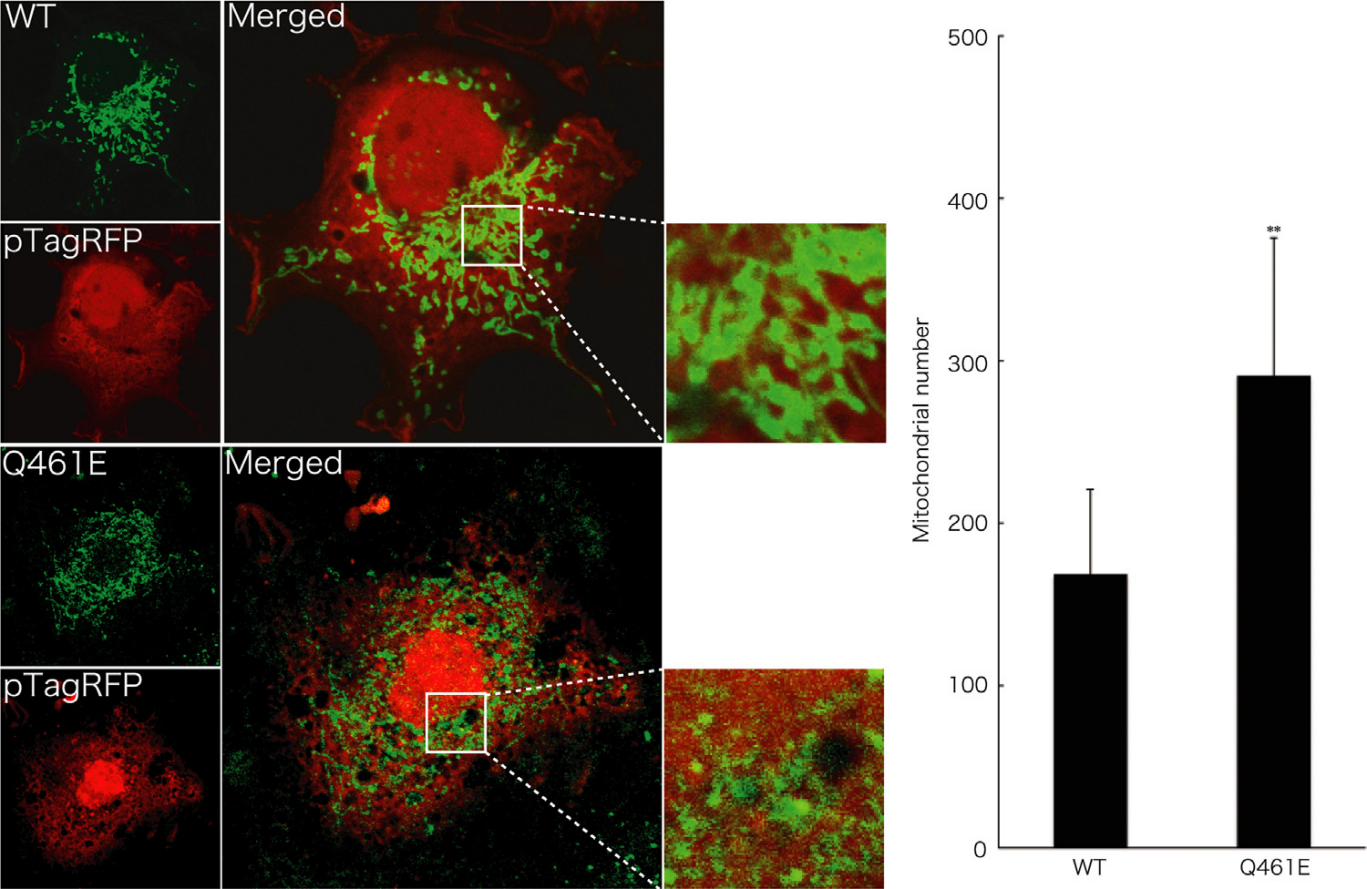
The number of mitochondria harboring HSPD1 (Q461E) is greater than that of mitochondria harboring wild type HSPD1. Cells were transfected with the empty vector pTagRFP (red fluorescence) together with the plasmid encoding wild type (WT) HSPD1 or HSPD1 (V98I) (green fluorescence). The number of mitochondria is also shown in the graph (**, *p*<0.05; *n*=12 cells from 3 independent experiments).

**Fig. 5:**
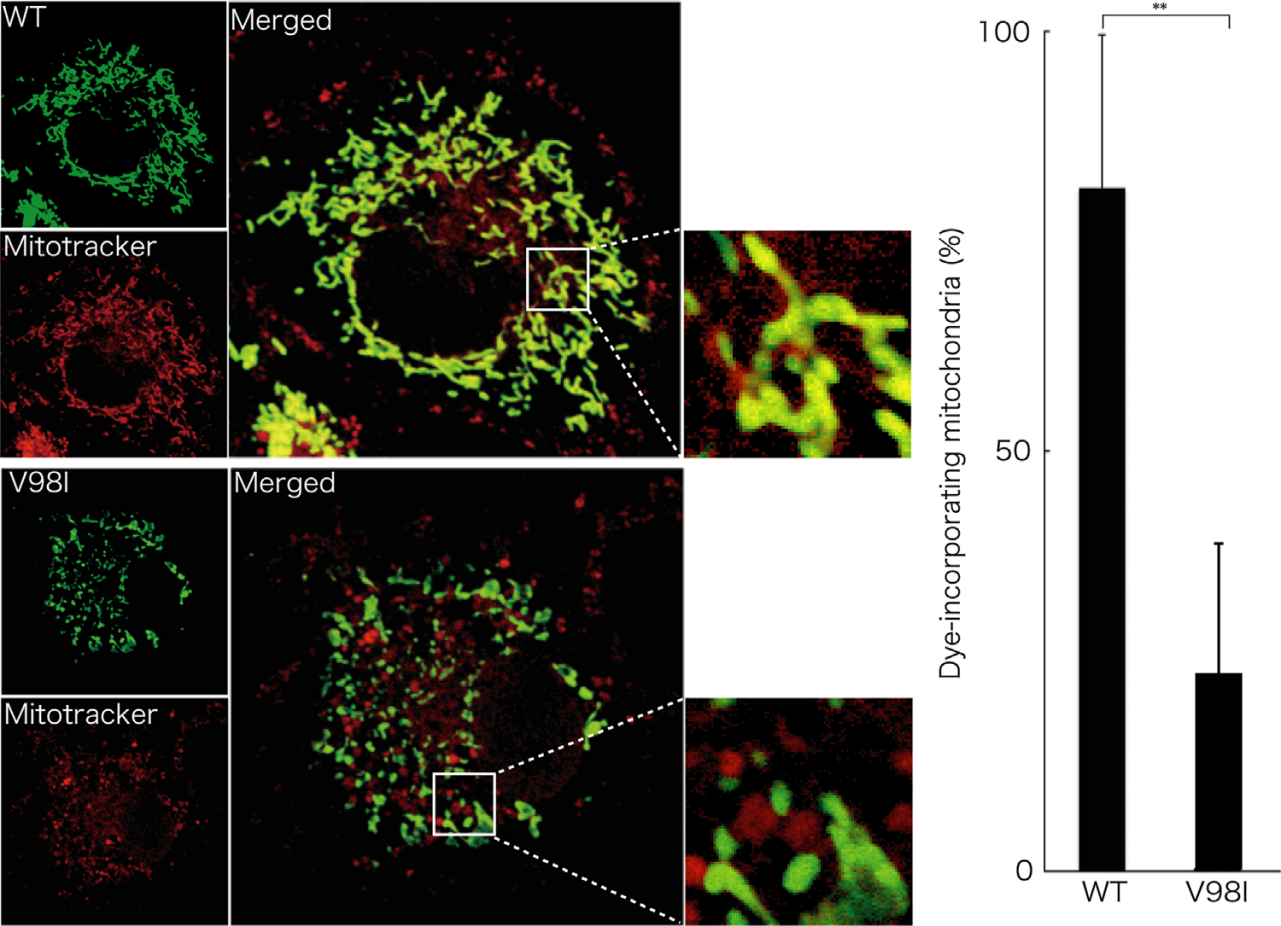
Mitochondria harboring HSPD1 (V98I) decrease MitoTracker Red CM-H2Xros dye-incorporating activity. Cells were transfected with the plasmid encoding wild type (WT) HSPD1 or HSPD1 (V98I) (green fluorescence) and were incubated with MitoTracker Red CM-H2Xros dye incorporated into active mitochondria (red fluorescence). The percentage of dye-incorporated mitochondria is shown in the graph (**, *p*<0.05; *n*=12 cells from 3 independent experiments).

**Fig. 6:**
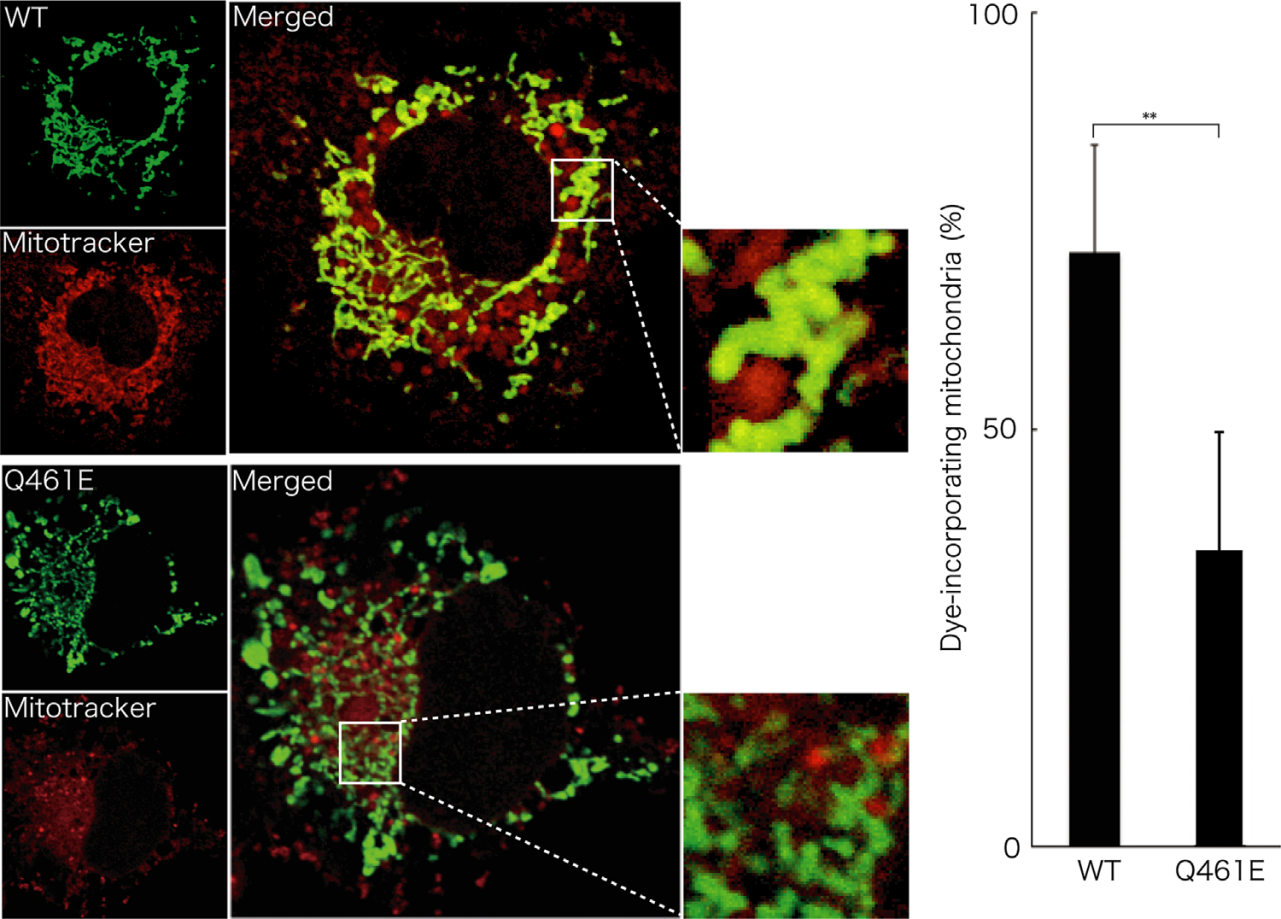
Mitochondria harboring HSPD1 (Q461E) decrease MitoTracker Red CM-H2Xros dye-incorporating activity. Cells were transfected with the plasmid encoding wild type (WT) HSPD1 or HSPD1 (V98I) (green fluorescence) and were incubated with MitoTracker Red CM-H2Xros dye incorporated into active mitochondria (red fluorescence). The percentage of dye-incorporated mitochondria is shown in the graph (**, *p*<0.05; *n*=12 cells from 3 independent experiments).

Before experiments, cells were treated with or without MitoTracker Red CM-H2Xros dye (Thermo Fisher Scientific). MitoTracker red fluorescent dye is accumulated in active mitochondria depending upon their membrane potential. Following culture medium removal, cells were immediately fixed using an Image-iT Fixation/Permeabilization kit (Thermo Fisher Scientific). The coverslips were mounted onto slides with Vectashield reagent (Vector Laboratories, Burlingame, CA, USA) for observation using confocal microscopy. The confocal images were collected using an IX81 microscope with a laser-scanning FV500 or FV1000 system (Olympus, Tokyo, Japan) and analyzed using FluoView software (Olympus) [3], [4]. In MitoTracker dye-incorporating assay, the respective pixel values of the mitochondrion-specific HSPD1’s green color and the yellow color merged with MitoTracker red fluorescent dye were measured using ImageJ software. The percentage of a merged yellow color value per a green color value was considered as that of MitoTracker dye-incorporating mitochondria. The length of mitochondria harboring HSPD1 (V98I) was shorter than that of mitochondria harboring wild type HSPD1 by ~30% (Fig. 1). Similar data was observed in HSPD1 (Q461E) (Fig. 2). As the result, the number of mitochondria harboring HSPD1 (V98I) or HSPD1 (Q461E) was ~1.5-fold greater than that of mitochondria harboring wild type HSPD1 and is depicted in Fig. 3, Fig. 4, respectively. Mitochondria harboring wild type HSPD1 had the ability to incorporate a typical quantity of MitoTracker dye. In contrast, HSPD1 (V98I) or HSPD1 (Q461E) decreased dye-incorporating activity by ~50% (Fig. 5, Fig. 6).

### 1.5 Statistical analysis

The values shown in the figure panels represent the means±standard deviation from separate experiments. Comparisons between two experimental groups were made using the unpaired Student’s *t* test (**, *p*<0.05). *p* Values less than 0.05 were considered significant.

## Conflict of interest

The authors declare that there is no conflict of interest.
